# Pirfenidone decreases mesothelioma cell proliferation and migration via inhibition of ERK and AKT and regulates mesothelioma tumor microenvironment *in vivo*

**DOI:** 10.1038/s41598-018-28297-x

**Published:** 2018-07-03

**Authors:** Chang Li, Veronika Rezov, Emmi Joensuu, Ville Vartiainen, Mikko Rönty, Miao Yin, Marjukka Myllärniemi, Katri Koli

**Affiliations:** 1 Research Programs Unit, Translational Cancer Biology, University of Helsinki, Helsinki, Finland; 2 Department of Thoracic and Cardiovascular Surgery, The First Affiliated Hospital of Soochow University, Medical College of Soochow University, Soochow, China; 3 Department of Pathology, University of Helsinki and Fimlab laboratories, Pathology, Tampere Finland; 4 University of Helsinki and Helsinki University Hospital, Heart and Lung Center and HUH diagnostics, Pulmonary Medicine, Helsinki, Finland

## Abstract

Malignant mesothelioma is an aggressive cancer with poor prognosis. It is characterized by prominent extracellular matrix, mesenchymal tumor cell phenotypes and chemoresistance. In this study, the ability of pirfenidone to alter mesothelioma cell proliferation and migration as well as mesothelioma tumor microenvironment was evaluated. Pirfenidone is an anti-fibrotic drug used in the treatment of idiopathic pulmonary fibrosis and has also anti-proliferative activities. Mesothelioma cell proliferation was decreased by pirfenidone alone or in combination with cisplatin. Pirfenidone also decreased significantly Transwell migration/invasion and 3D collagen invasion. This was associated with increased BMP pathway activity, decreased *GREM1* expression and downregulation of MAPK/ERK and AKT/mTOR signaling. The canonical Smad-mediated TGF-β signaling was not affected by pirfenidone. However, pirfenidone blocked TGF-β induced upregulation of ERK and AKT pathways. Treatment of mice harboring mesothelioma xenografts with pirfenidone alone did not reduce tumor proliferation *in vivo*. However, pirfenidone modified the tumor microenvironment by reducing the expression of extracellular matrix associated genes. In addition, *GREM1* expression was downregulated by pirfenidone *in vivo*. By reducing two major upregulated pathways in mesothelioma and by targeting tumor cells and the microenvironment pirfenidone may present a novel anti-fibrotic and anti-cancer adjuvant therapy for mesothelioma.

## Introduction

Malignant mesothelioma is an asbestos exposure-related aggressive cancer, which most often occurs in the pleura^1,2^. The prognosis is poor, survival is usually only ~10 months from diagnosis. Mesothelioma originates from serosal mesothelial cells with three histological subtypes. The epithelioid mesothelioma is the most common type with prominent papillo-tubular structures, the sarcomatoid mesothelioma is characterized by spindled cells mimicking fibrosarcoma and the biphasic mesothelioma displays both epithelioid and sarcomatoid histology. Sarcomatoid mesothelioma is the most aggressive type and has the worst prognosis^3^. Mesothelioma shows progressive local growth and aggressive invasion into the neighboring tissue. Tumor cells can also travel to nearby lymph nodes, but the tendency to distant metastasis is usually low. Currently, mesothelioma remains an incurable disease with limited treatment options^4,5^.

The genetic and molecular profiling of mesothelioma has led to the identification of pathways involved in driving mesothelioma growth and invasion. Mesothelioma is characterized by loss of tumor suppressor genes rather than oncogene activation. The most commonly mutated genes are *CDKN2A/ARF*, *BAP1* and *NF2*^6^. Analyses of alterations in signaling pathway activities have identified consistent upregulation of the PI3K/AKT/mTOR pathway^7^. In addition, activation of epidermal growth factor (EGF), platelet derived growth factor (PDGF) and hepatocyte growth factor (HGF) pathway signaling as well as the angiogenic vascular endothelial growth factor (VEGF) and fibroblast growth factor (FGF) pathways have been reported^8,9^. We and others have linked transforming growth factor (TGF)-β pathway activation to mesothelioma invasive growth^8,10,11^. The characterization of these re-expressed developmental pathways, which drive proliferation and give rise to migratory and invasive cancer cell phenotypes, may lead to new target identification.

We have recently identified the BMP inhibitor protein gremlin-1 to be upregulated in mesothelioma^12^. Gremlin-1 drives a mesenchymal and chemoresistant phenotype in mesothelioma cells. Gremlin-1 expression is also associated with invasion and metastasis as well as tumor vascularization in an *in vivo* mouse model^11^. Downregulation of BMP pathway activity is associated with increased TGF-β pathway activity, resulting in imbalance of growth factor activities. Idiopathic pulmonary fibrosis (IPF) is an interstitial lung disease characterized by accumulation of fibroblasts/myofibroblasts, excessive matrix production and altered TGF-β/BMP signaling balance^13,14^. We have shown that restoration of the impaired BMP signaling activity, by administration of BMP-7 or using the small molecule drug tilorone, reduces fibrosis in experimental mouse models^15,16^. Fibrotic alterations in the tumor microenvironment can promote tumor proliferation and invasive behavior^17^. Pirfenidone is an anti-fibrotic drug used in the treatment of IPF patients^13^. Although the mechanisms of action are not fully characterized, pirfenidone is thought to act by reducing TGF-β-mediating signaling in IPF^18^. We hypothesized that by altering tumorigenic signaling pathways in mesothelioma cells and by modulation of the tumor stroma pirfenidone could reduce mesothelioma cell growth and invasion.

## Results

### Pirfenidone reduces mesothelioma cell proliferation, migration and 3D invasive growth

**Figure 1:**
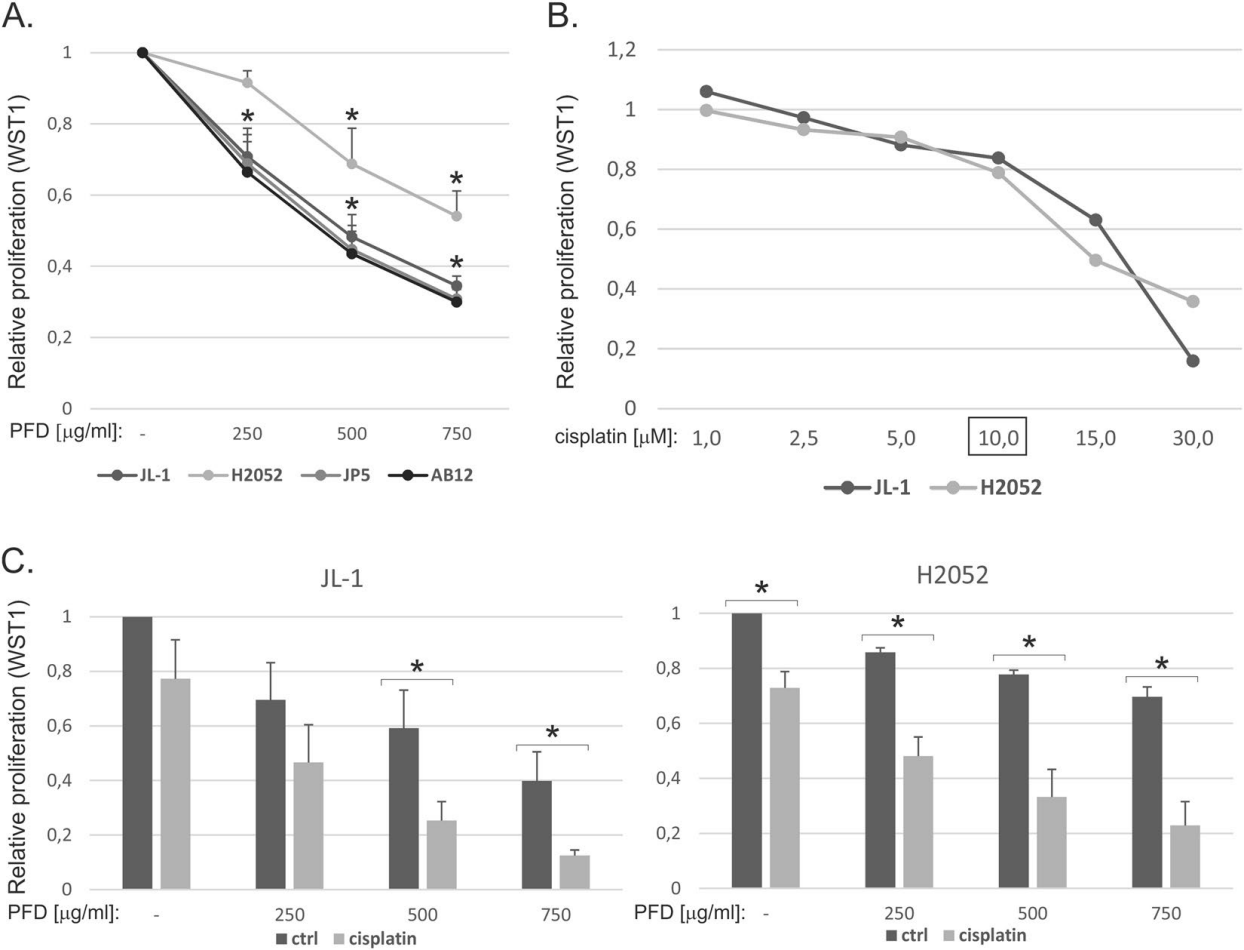
Pirfenidone inhibits mesothelioma cell proliferation. WST-1 assay was used to analyze cell proliferation. (**A**) Human (JL-1, H2052 and JP5) and mouse (AB12) mesothelioma cells were treated with increasing concentrations of pirfenidone (PFD, 0–750 μg/ml) for 2 days. The results are expressed relative to the proliferation in control treated cells, which was set to 1. The error bars represent SD (n = 3). *p < 0.05. (**B**) JL-1 and H2052 cells were treated with increasing concentrations of cisplatin (0–30 μM) for 2 days. The results are expressed relative to the proliferation in control treated cells, which was set to 1. A representative experiment is shown. For combined treatment studies with pirfenidone a concentration of 10 μM cisplatin was chosen. (**C**) JL-1 and H2052 cells were treated with cisplatin and/or pirfenidone (PFD, 0–750 μg/ml) for 2 days. The results are expressed relative to the proliferation in control treated cells, which was set to 1. The error bars represent SD (n = 3). *p < 0.05.

First, we analyzed the effects of pirfenidone on mesothelioma cell proliferation. JL-1, H2052 and JP5 human mesothelioma cells as well as AB12 mouse mesothelioma cells showed significantly reduced proliferation when treated with pirfenidone for 48 hours (Fig. 1A). Pirfenidone concentration of 750 μg/ml reduced proliferation in H2052 cells to ~50% of control level (non-treated cells), while in the other cells ~70% reduction was observed. A concentration of 10 μM cisplatin reduced proliferation ~20% in JL-1 and H2052 cells (Fig. 1B) and was chosen for further experiments combining cisplatin with pirfenidone. Cisplatin had at least an additive effect in the reduction of JL-1 and H2052 mesothelioma cell proliferation when combined with pirfenidone (Fig. 1C). Cisplatin and pemetrexed combination presents the standard chemotherapy regiment in the treatment of mesothelioma patients^19^. We tested also pemetrexed in a similar proliferation assay, but did not find any additional effect when combined with pirfenidone (data not shown).

**Figure 2:**
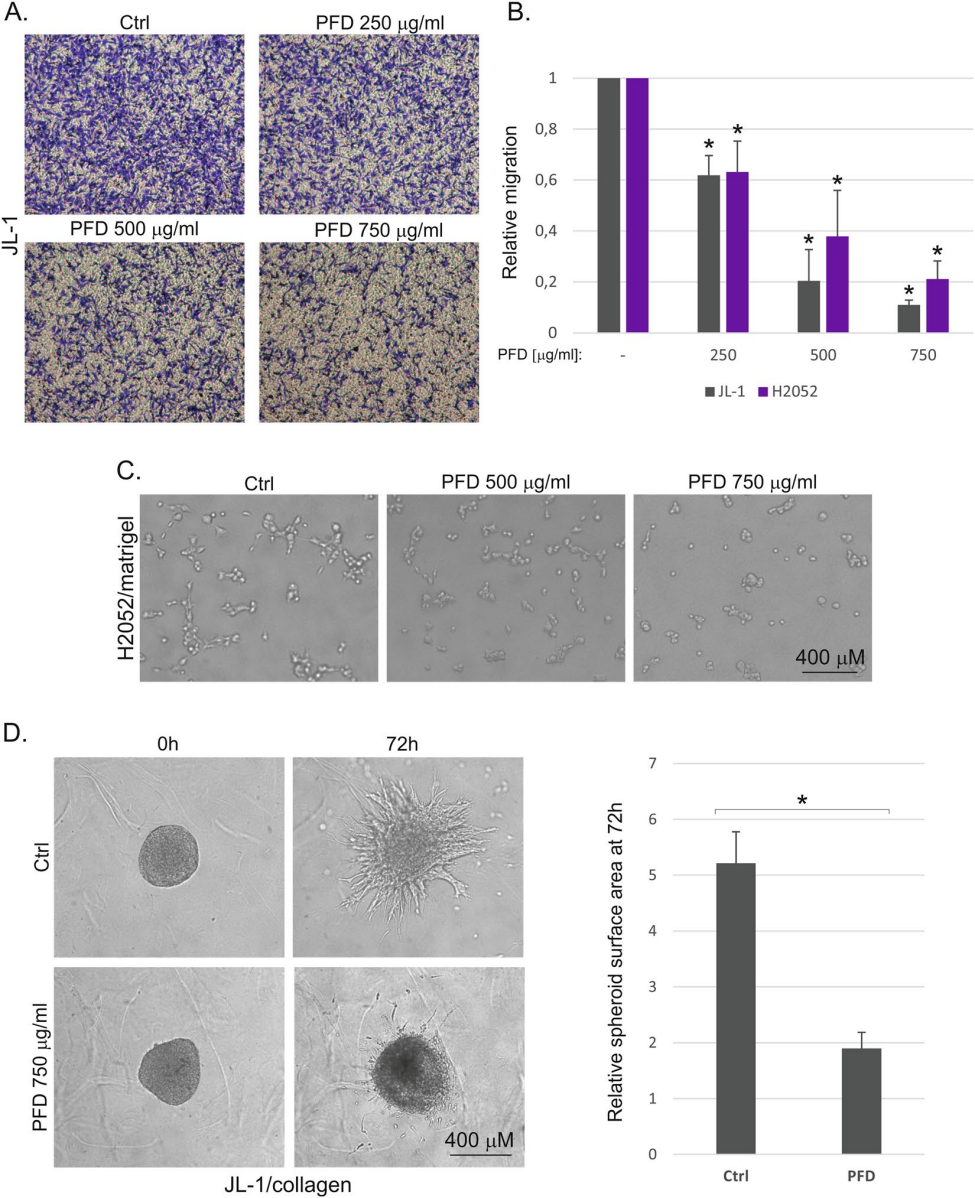
Pirfenidone reduces mesothelioma cell migration and 3D invasive growth. (**A**) Invasive migration was analyzed using collagen 1 coated Transwell inserts. Control or pirfenidone (PFD) treated migrated cells were fixed, stained and imaged 16 hours after seeding. Representative images of crystal violet stained JL-1 cells are shown. (**B**) Invasive migration of JL-1 and H2052 cells 16 hours after seeding is shown. Graphs represent quantification of relative migration. The error bars represent SD (n = 3). *p < 0.05. (**C**) H2052 cells were embedded into 3D Matrigel matrix. Images of control and pirfenidone (PFD) treated cells from a representative experiment are shown. (**D**) Invasive growth of control or pirfenidone (PFD) treated JL-1 cells was analyzed in 3D collagen 1 matrix. Cells were followed up to 72 hours. Representative images are shown. Graph shows quantification as relative spheroid surface area. The error bars represent SD (n = 3). *p < 0.05.

Transwell migration assay was used to assess the effect of pirfenidone on mesothelioma cell migration. JL-1 and H2052 cell migration/invasion through collagen 1 coated inserts was reduced significantly in a concentration dependent manner (Fig. 2A,B). H2052 cells are able to invade and sprout through the surrounding matrix when embedded into 3D Matrigel^11^. Pirfenidone reduced sprouting of H2052 cells in 3D Matrigel (Fig. 2C). Invasive growth and sprouting of JL-1 cells in 3D collagen matrix was also noticeably reduced by pirfenidone treatment (Fig. 2D and Supplementary Fig. 2). These results suggest that pirfenidone is a novel inhibitor of mesothelioma cell proliferation and migration.

### BMP signaling activity is increased in pirfenidone treated mesothelioma cells

**Figure 3:**
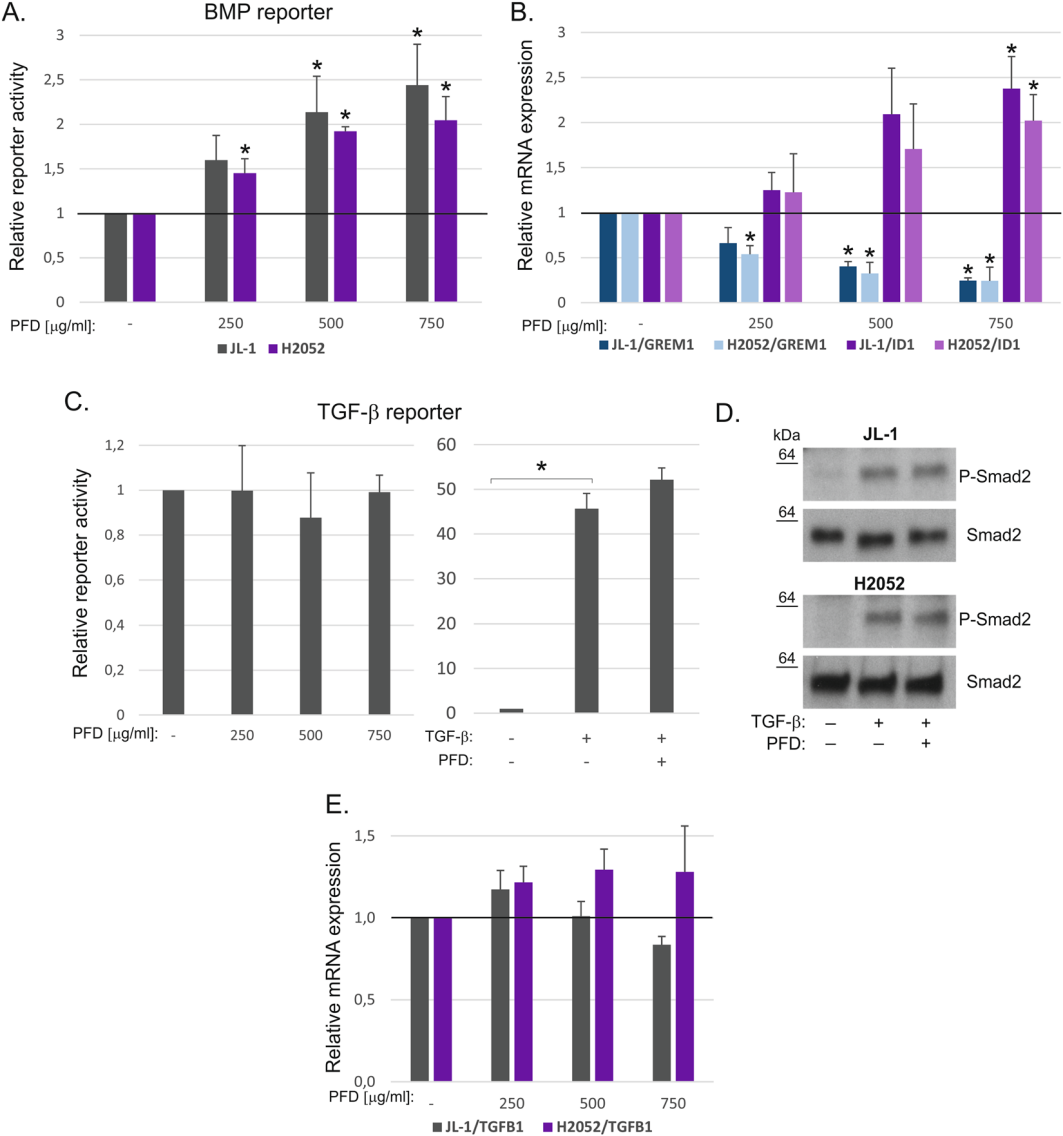
Increased BMP pathway activity in pirfenidone treated cells. (**A**) BMP [(Bre)_2_-luc] pathway activity was analyzed using a promoter reporter assay. Promoter activities in pirfenidone (PFD) treated cells are shown relative to the level in each control (JL-1 or H2052), which was set to 1. The error bars represent SD (n = 3). *p < 0.05. (**B**) Expression of *ID1* and *GREM1* genes in pirfenidone (PFD) treated JL-1 and H2052 cells. The results are expressed relative to each control, which was set to 1. The error bars represent SD (n = 3). *p < 0.05. (**C**) TGF-β [(CAGA)_12_-luc] pathway activity was analyzed in H2052 cells using a promoter reporter assay. Promoter activities in pirfenidone (PFD, 750 μg/ml) and/or TGF-β1 (0.5 ng/ml) treated cells are shown relative to the level in control, which was set to 1. (**D**) Pirfenidone (PFD, 750 μg/ml) and TGF-β1 (0.5 ng/ml) treated JL-1 and H2052 cell lysates were analyzed by Western blotting using antibodies specific to P-Smad2 and Smad2. The molecular weight markers (kDa) are shown on the left. The western blots were derived under the same experimental conditions from the same cell lysate of each treatment group (JL-1 and H2052). (**E**) Expression of *TGFB1* gene in pirfenidone (PFD) treated JL-1 and H2052 cells. The results are expressed relative to each control, which was set to 1. The error bars represent SD (n = 3).

We have previously linked high gremlin-1 expression and aberrant TGF-β/BMP signaling activity to mesothelioma invasive growth^11^. Gremlin-1 mediated reduction in BMP-pathway activity and increased TGF-β pathway activity drive invasive growth of mesothelioma cells^11^. Here, we observed a concentration-dependent increase in BMP-pathway activity in pirfenidone treated JL-1 and H2052 cells (Fig. 3A). This was associated with increased expression of the BMP-pathway target gene *ID1* (Fig. 3B). Interestingly, the expression of *GREM1* was significantly reduced by pirfenidone in the two different mesothelioma cell lines, suggesting a mechanism by which pirfenidone can rescue BMP-pathway activity (Fig. 3B). TGF-β-pathway reporter activity (Smad3 dependent, see Methods) was not reduced by pirfenidone with or without TGF-β treatment (Fig. 3C). TGF-β induced phosphorylation of Smad2 was not reduced either in JL-1 or H2052 cells (Fig. 3D). Furthermore, no significant changes were noted in *TGFB1* mRNA expression levels (Fig. 3E). In contrast to previous observations, these results suggest that at least in mesothelioma cells, the anti-tumor and anti-fibrotic activity mediated by pirfenidone is independent of the canonical TGF-β signaling.

### MAPK/ERK and AKT pathway activities are reduced by pirfenidone

**Figure 4:**
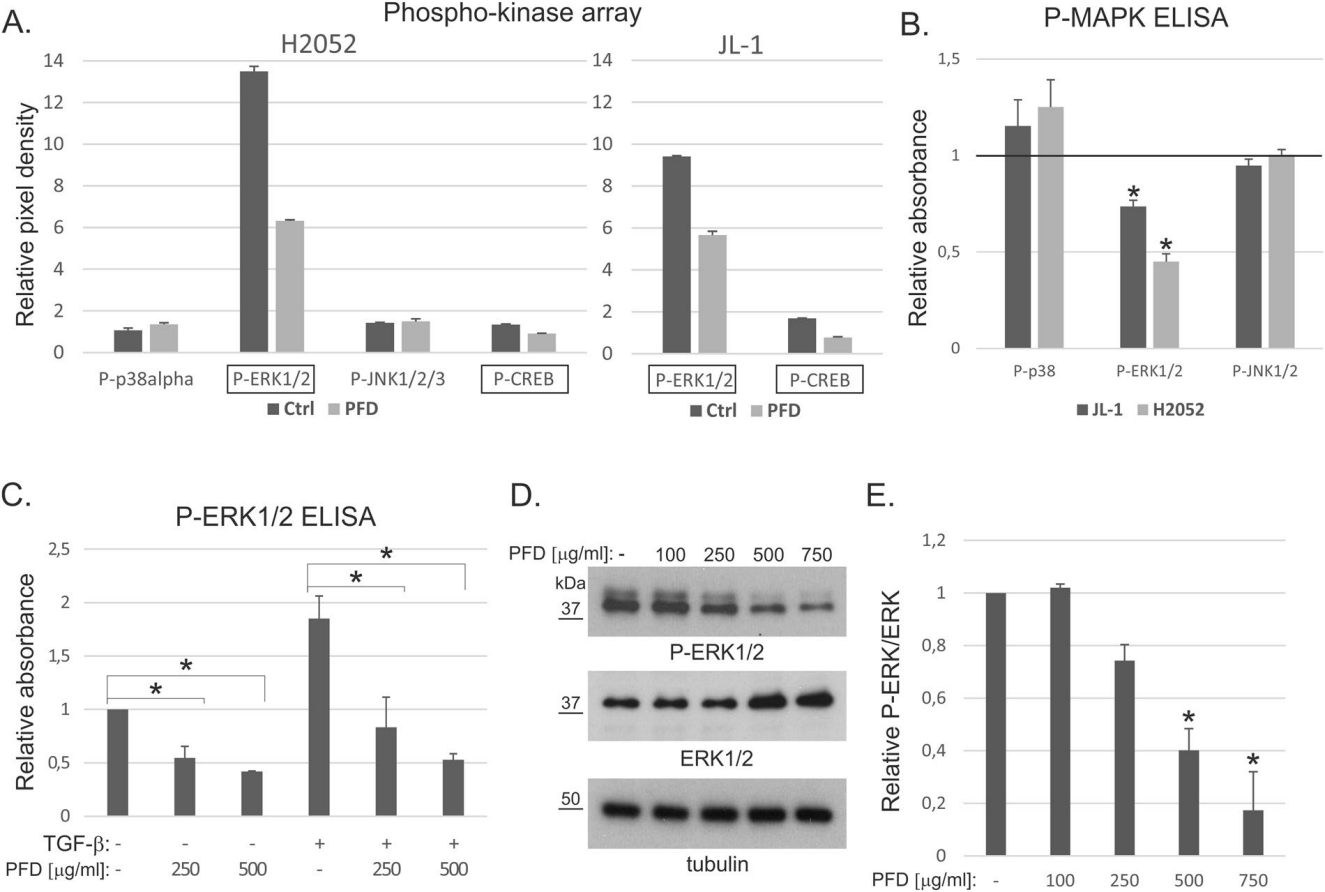
Pirfenidone reduces ERK activation. (**A**) JL-1 and H2052 cell lysates from control and pirfenidone (PFD, 500 µg/ml, 4 hours) treated cells were analyzed using a commercial phospho-protein array. Quantification of alterations in the amounts of phospho-proteins (n = 2) is shown. (**B**) JL-1 and H2052 cell lysates from pirfenidone (PFD, 500 µg/ml, 4 hours) treated cells were analyzed by P-MAPK ELISA. The results are expressed relative to each control, which was set to 1. The error bars represent SD (n = 3). *p < 0.05. (**C**) H2052 cell lysates from pirfenidone (PFD, 500 µg/ml) and/or TGF-β1 (0.5 ng/ml) treated (4 hours) cells were analyzed by P-ERK1/2 ELISA. The results are expressed relative to control, which was set to 1. The error bars represent SD (n = 3). *p < 0.05. (**D**) Pirfenidone (PFD) treated H2052 cell lysates were analyzed by Western blotting using antibodies specific to P-ERK1/2, ERK1/2 or tubulin. The molecular weight markers (kDa) are shown on the left. The western blots were derived under the same experimental conditions from the same cell lysate. (**E**) Quantification of band intensities. P-ERK/ERK levels were calculated and normalized using the tubulin loading control. The results are expressed relative to control, which was set to 1. The error bars represent SD (n = 3). *p < 0.05.

To investigate cellular signaling pathways affected by pirfenidone treatment, we analyzed alterations in phospho-kinase levels in JL-1 and H2052 cells using a commercial array (see Methods). The assay was repeated twice to screen for signaling differences. Phospho-ERK1/2 (P-ERK1/2) and P-CREB were the only phospho-proteins consistently reduced by pirfenidone treatment in both cell lines (Fig. 4A). P-ERK1/2 levels were also analyzed by ELISA and observed to be decreased by pirfenidone, while P-p38 and P-JNK1/2 levels were not altered (Fig. 4B). Since TGF-β is known to regulate MAPK-pathways^20^ we analyzed P-ERK1/2 levels also in TGF-β stimulated cells by ELISA. TGF-β was found to increase P-ERK1/2 levels in H2052 cells (Fig. 3C). Pirfenidone reduced P-ERK1/2 in both TGF-β treated and non-treated cells. In agreement, immunoblotting analysis of P-ERK1/2 levels indicated significant concentration dependent reduction by pirfenidone in H2052 cells (Fig. 4D,E). Although pirfenidone did not reduce canonical Smad-mediated TGF-β signaling in mesothelioma cells, it was able to reduce TGF-β-mediated induction of ERK-pathway activation.

**Figure 5:**
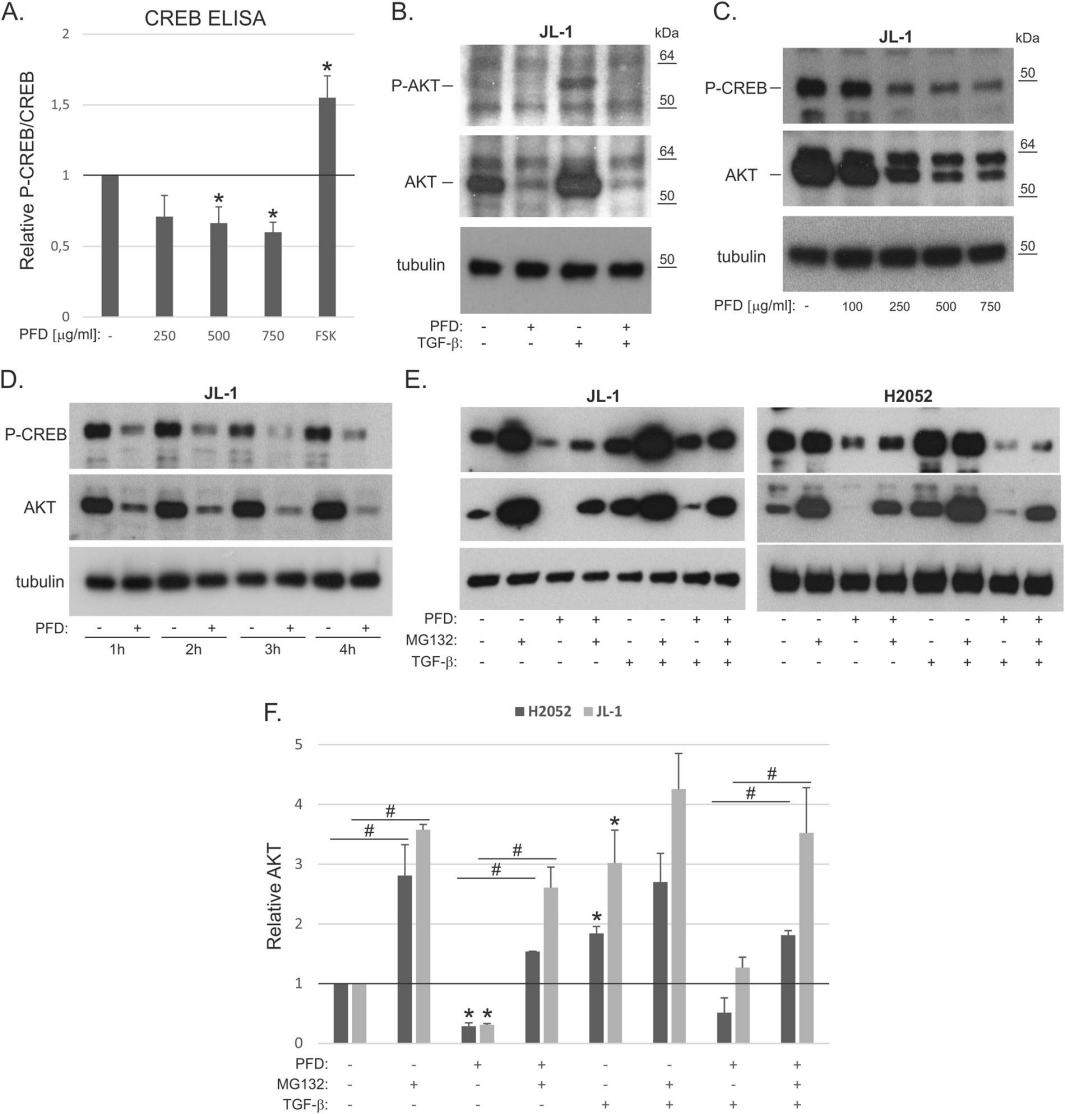
CREB and AKT pathway regulation by pirfenidone. (**A**) JL-1 cell lysates from pirfenidone (PFD, 4 hours) or forskolin (FSK, 10 µM, positive control) treated cells were analyzed by CREB ELISA. P-CREB/CREB results are expressed relative to control, which was set to 1. The error bars represent SD (n = 3). *p < 0.05. (**B**) Cell lysates from pirfenidone (PFD, 750 µg/ml) and/or TGF-β1 (0.5 ng/ml) treated (4 hours) cells were analyzed by Western blotting using antibodies specific to P-AKT, AKT or tubulin. The molecular weight markers (kDa) are shown on the right. (**C**,**D**) Cell lysates from pirfenidone (PFD, 0–750 µg/ml) treated (1–4 hours) cells were analyzed by Western blotting using antibodies specific to P-CREB, AKT or tubulin. (**E**) Cell lysates from pirfenidone (PFD, 750 µg/ml), MG132 (10 µM) and/or TGF-β1 (0.5 ng/ml) treated (4 hours) cells were analyzed by Western blotting using antibodies specific to P-CREB, AKT or tubulin. (**F**) Quantification of AKT band intensities, which were normalized using the tubulin loading control. The results are expressed relative to each control (JL-1 or H2052), which was set to 1. The error bars represent SD (n = 3). *p < 0.05 when compared to the sample without any treatment. ^#^p < 0.05. At least three independent experiments were performed for all quantifications. The western blots were derived under the same experimental conditions from the same cell lysate of each treatment group (**B**–**E**). The original full-length western blot images (**D**) and additional exposures (**B**) are shown in Supplementary figure 5.

AKT-mediated signaling is often activated in mesothelioma and contributes to cell survival and proliferation^7^. It is also known to be regulated by TGF-β pathway activity^21^. The effect of pirfenidone on AKT and CREB proteins was therefore analyzed using ELISA and Western blotting. P-CREB levels were decreased by pirfenidone and increased by forskolin (FSK) treatment used as a positive control for CREB activation (Fig. 5A). Total CREB levels were not altered by pirfenidone or forskolin in the ELISA assay (not shown). Western blotting indicated that P-AKT level was low in non-treated JL-1 cells and was increased by TGF-β treatment (Fig. 5B). Pirfenidone reduced P-AKT level in TGF-β treated cells. Notably, total AKT level was also decreased in both TGF-β treated and non-treated cells. A concomitant decrease in P-CREB levels was observed, consistent with the ELISA results (Fig. 5C). Pirfenidone induced decrease in P-CREB and AKT levels was concentration- and time-dependent (Fig. 5C,D). In JL-1 cells 1 hour treatment with pirfenidone was enough to reduce AKT level suggesting that protein degradation may mediate the effect of pirfenidone. Treatment of JL-1 and H2052 cells with the proteasome inhibitor MG132 (10 μM) increased AKT protein levels in TGF-β treated and non-treated cells (Fig. 5E,F). In addition, MG132 rescued AKT levels in pirfenidone treated cells. P-CREB levels also increased in TGF-β treated and non-treated JL-1 cells, however, MG132 was only slightly able to rescue P-CREB levels in pirfenidone treated JL-1 or H2052 cells (Fig. 5E,F, Supplementary Fig. 3). Similar to what was found in TGF-β treated cells, pirfenidone decreased AKT levels also in the presence of PDGF-BB (data not shown). The results indicate that pirfenidone can decrease AKT and CREB pathways and that proteasome mediated degradation of AKT may play a functional role in mesothelioma cells.

### Pirfenidone decreases p70S6K phosphorylation

**Figure 6:**
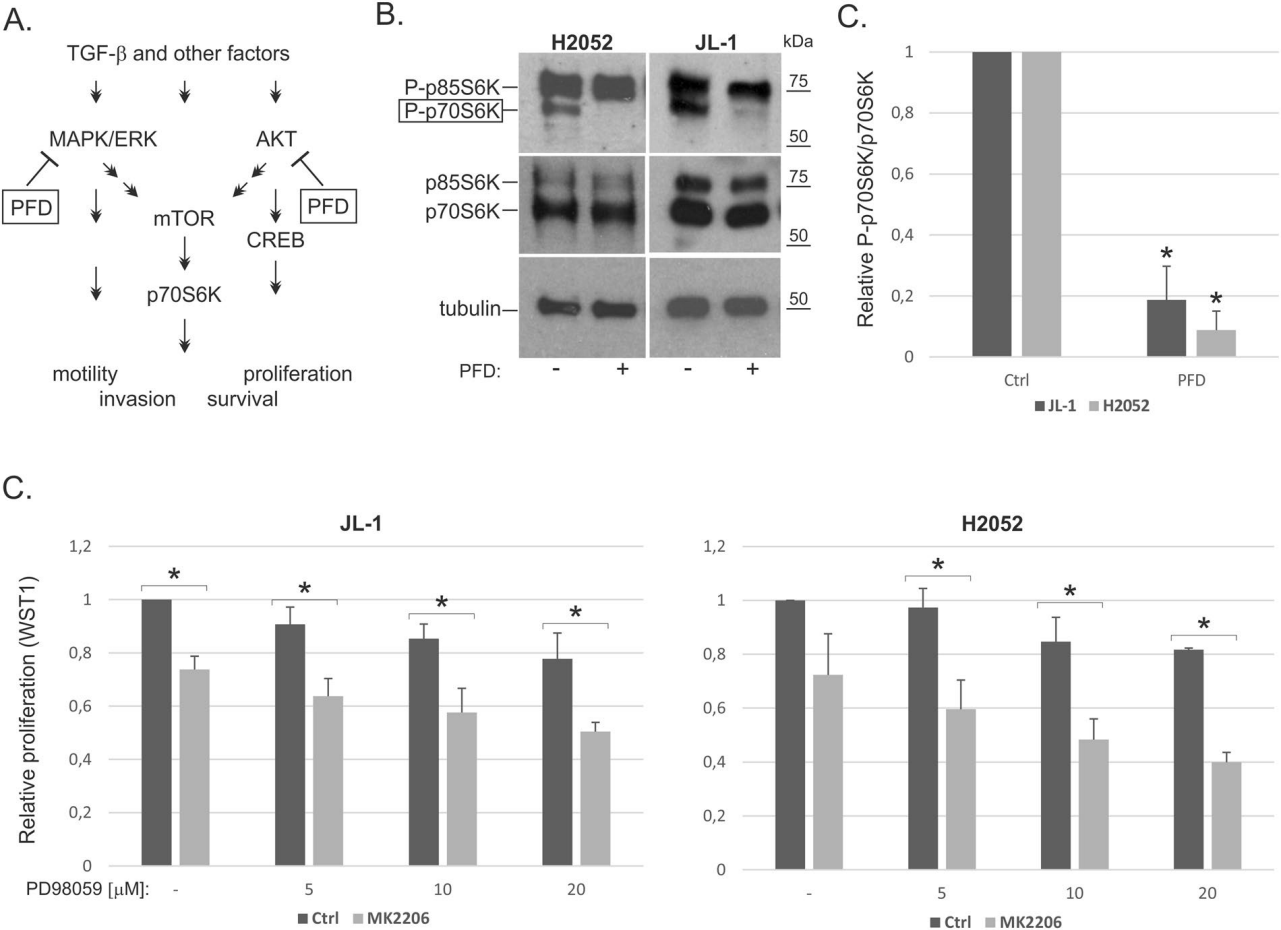
p70S6K phosphorylation is decreased by pirfenidone (**A**). A schematic presentation of pirfenidone activity. (**B**) Cell lysates from pirfenidone (PFD, 750 µg/ml) treated (4 hours) cells were analyzed by Western blotting using antibodies specific to P-p70S6K/P-p85S6K, p70S6K/p85S6K or tubulin. (**F**) Quantification of P-p70S6K and p70S6K band intensities. P-p70S6K/p70S6K levels were calculated and the results are expressed relative to each control (JL-1 or H2052), which was set to 1. The error bars represent SD (n = 3–4). *p < 0.05. At least three independent experiments were performed for all quantifications. The western blots were derived under the same experimental conditions from the same cell lysate of each treatment group (JL-1 and H2052).

Activation of the PI3K/AKT pathway leads to activation of mammalian target of rapamycin (mTOR), which initiates phosphorylation and activation of p70S6K (Fig. 6A). Pirfenidone treatment was found to decrease P-p70S6K levels in both JL-1 and H2052 cells (Fig. 6B,C). Taken together, our results indicate that pirfenidone targets multiple pathways (MAPK/ERK and AKT/mTOR) involved in mesothelioma cell survival and motility (Fig. 6A). Consistent with this observation, the effect of pirfenidone on mesothelioma cell proliferation was replicated by combining the AKT inhibitor (MK2206) and the MEK inhibitor (PD98059). Proliferation was decreased in an additive manner (Fig. 6D).

### Pirfenidone modulates mesothelioma tumor microenvironment

**Figure 7:**
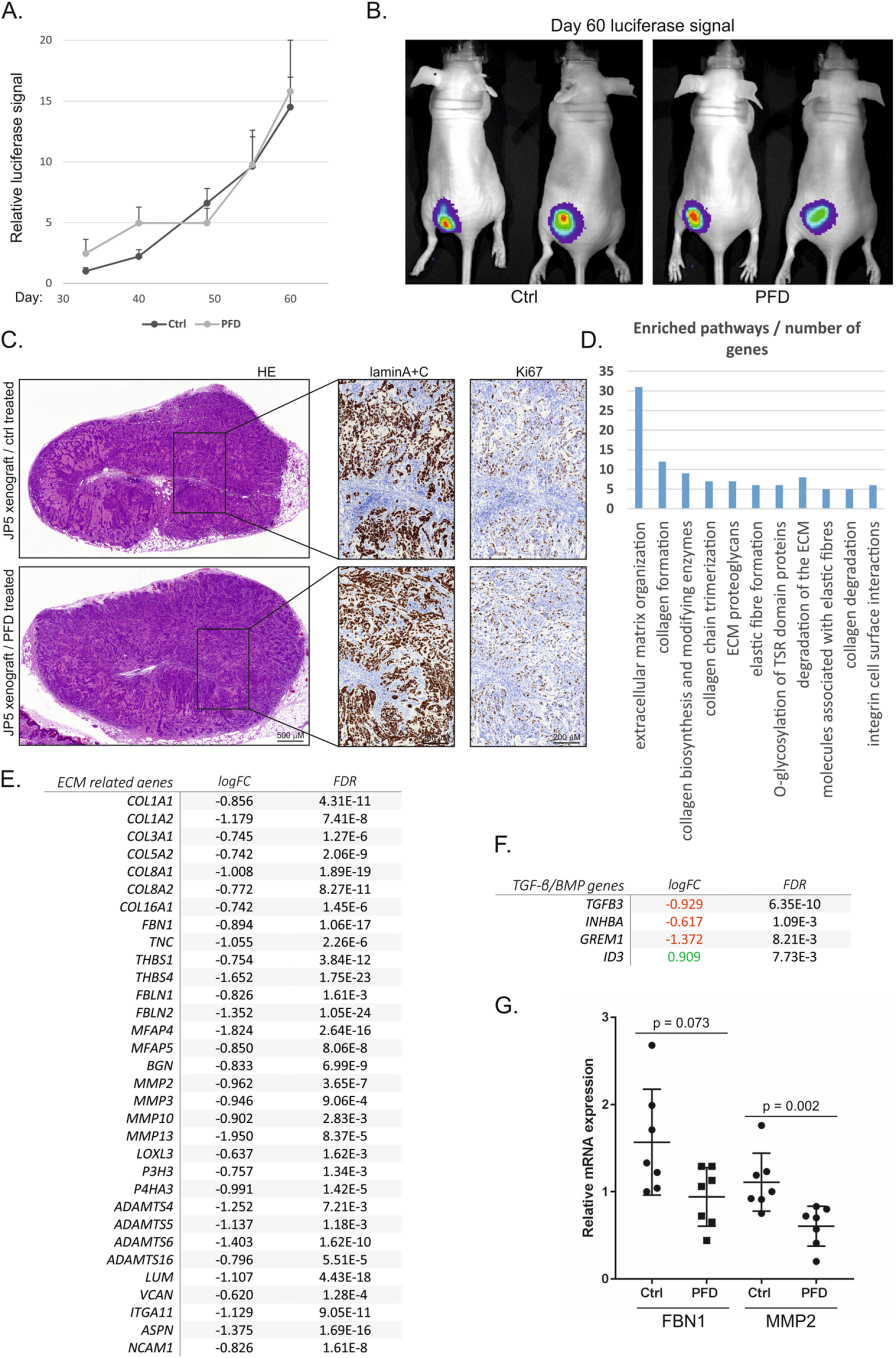
Pirfenidone induces downregulation of the expression of ECM associated genes *in vivo* (**A**). JP5 mesothelioma cells transduced to express a luciferase marker were injected subcutaneously as Matrigel plugs. Tumor growth was followed by luciferase signal measurements for 60 days. The error bars represent SEM (n = 8). (**B**) Images of luciferase signal detection of control and pirfenidone (PFD, 200 mg/kg/day) treated mice at day 60. (**C**) Subcutaneous tumors from control and pirfenidone (PFD) treated mice were stained with hematoxylin & eosin (HE), antibodies specific to human lamin A + C or the proliferation marker Ki67. Representative images are shown. (**D**) RNA sequencing was performed from Ctrl (n = 3) and pirfenidone (n = 4) treated tumors. Gene set enrichment analysis was performed using Reactome. Top hit pathways are shown. (**E**) A list of genes included in the “extracellular matrix organization” Reactome-pathway. Logarithmic fold change (logFC) and false discovery rate (FDR) for each gene are shown. (**F**) Differentially expressed TGF-β/BMP pathway genes. (**G**) Expression of *FBN1* and *MMP2* genes in the tumor tissue of control (Ctrl) and pirfenidone (PFD) treated mice (n = 7). Quantitative RT-PCR results are expressed relative to control tumor-1, which was set to 1.

An *in vivo* study was performed using injection of JP5 mesothelioma cells subcutaneously as Matrigel plugs into nude mice (see Methods). In agreement with our previous studies all mice developed tumors as detected by luciferase signal^11^. Treatment with pirfenidone (200 mg/kg/day PO) was started one day after the injection of the tumor cells. Mice receiving pirfenidone showed a non-significant weight loss from day 12 onwards (Supplementary Fig. 4). No difference in tumor luciferase signal was detected between control (PBS) and pirfenidone treated mice during the experiment (n = 8, Fig. 7A,B). Mice were sacrificed at 2 months and the tumors stained with laminA + C antibody to identify cells of human origin. Lamin staining as well as tissue histology revealed an epithelioid pattern as expected (Fig. 7C). Ki67 staining indicated no significant differences in proliferation between control and pirfenidone treated mice [percent Ki67 positive area (mean ± SD) was 12.1 ± 1.7 in control and 12.2 ± 2.1 in pirfenidone group, n = 8]. The results suggest that pirfenidone as a single agent at 200 mg/kg dose is not enough to reduce tumor cell proliferation.

To gain insight into pirfenidone induced alterations in gene expression, RNA sequencing of tumor tissue samples was performed (see Methods). Differentially expressed genes were mostly genes downregulated in pirfenidone treated tumors (101/127, Supplementary Table 1). Analysis of enriched Reactome pathways identified an extracellular matrix organization gene signature with over 30 genes downregulated by pirfenidone treatment (Fig. 7D). This gene signature included collagen genes (e.g. *COL1A1*, *COL1A2*, *COL3A1*), collagen modifying genes (e.g. *LOXL3*, *P3H3*, *P4HA3*), other matrix protein and glycoprotein genes (e.g. *FBN1*, *TNC*, *THBS1*, *FBLN1*, *LUM*, *VCAN*) as well as matrix modifying proteolytic enzymes (e.g. *MMP2*, *MMP3*, *ADAMTS4*, Fig. 7E). Consistent with *in vitro* results pirfenidone also reduced *GREM1* expression and induced BMP pathway target gene *ID3* expression *in vivo* (Fig. 7F). In addition, *TGFB3* and *INHBA* expression was decreased. We have previously shown high expression of *FBN1* and *FBN2* as well as *MMP2* in association with high *GREM1* expression in JP5 cells as well as in other mesothelioma cells^11,12^. Quantitative RT-PCR analyses confirmed that *MMP2* expression was significantly decreased in pirfenidone treated tumors (Fig. 7G). In addition, *FBN1* showed a trend (p = 0.073) towards decreased expression. *GREM1* levels were low and not reliably detectable by RT-PCR. We and others have shown that many of the differentially expressed genes identified by RNA sequencing are linked to a mesenchymal and chemoresistant phenotype in mesothelioma suggesting that pirfenidone treatment can alter the fibrotic tumor microenvironment.

## Discussion

Mesothelioma is a cancer characterized by aggressive growth and local invasion. Mesothelioma originates from serosal mesothelial cells, which are mesodermal derived cells displaying both epithelial and mesenchymal characteristics^22^. Mesothelioma cells often acquire a mesenchymal, migratory and chemoresistant phenotype^12,23^. The cells are embedded in a fibrotic, matrix rich microenvironment, which further favors resistance to chemotherapy. Growth factor activities such as BMP and TGF-β signaling pathways have been shown to regulate mesothelioma microenvironment and invasive growth^1,12^. TGF-β/activin pathways are increased in mesothelioma tumor tissue as well as in primary mesothelioma cells^10,11^. Activin-A was recently identified as a novel prognostic biomarker in epithelioid type mesothelioma^24^. We have shown that upregulation of gremlin-1 and modulation of BMP pathway activity is linked to invasion and vascularization in experimental mesothelioma xenograft tumors^11^. The TGF-β/BMP signaling balance is aberrantly regulated in mesothelioma similar to what we have observed in idiopathic pulmonary fibrosis (IPF), which is also characterized by connective tissue accumulation and migratory mesenchymal cell phenotypes^14,15^.

Repurposing of anti-fibrotic drugs to cancer treatment is not a novel idea and should be fully explored, especially to aggressive cancers with ECM rich microenvironment such as mesothelioma. Pirfenidone is a synthetic pyridone compound approved for the treatment of IPF first in Japan and later in Europe (2011) and US (2014). It has been shown to decrease fibroblast proliferation, ECM accumulation as well as to reduce inflammatory cell activity in the fibrotic lung^25,26^. In the present study, pirfenidone was found to significantly decrease mesothelioma cell proliferation. We also observed increased activity when combined with cisplatin but not with pemetrexed. This is in agreement with a recent study where pirfenidone and cisplatin in combination was shown to increase cell death in non-small cell lung cancer cells as well as in lung derived cancer-associated fibroblasts^27^. Mesothelioma cell migration/invasion through collagen matrix as well as invasive growth into 3D collagen were also found decreased by pirfenidone treatment. In agreement, a study by Surolia *et al*.^28^ indicated that in 3D spheroids cultured from IPF patient lung biopsy cells pirfenidone can reduce invasiveness. Pirfenidone was also able to reduce pancreatic stellate cell proliferation and migration/invasion through Matrigel matrix^29^. However, invasiveness through collagen matrix mimics better the microenvironment in the mesothelioma tumor tissue. Consistent with these events being regulated by TGF-β/BMP signaling activities, we observed increased BMP pathway activity and decreased gremlin-1 expression in pirfenidone treated mesothelioma cells.

The anti-fibrotic and anti-inflammatory mechanisms of action of pirfenidone are not fully characterized. It is mainly suggested to function through inhibition of TGF-β expression and/or TGF-β induced signal transduction pathways^30,31^. Pirfenidone modulates the properties of IPF fibroblasts and myofibroblasts by reducing proliferation and myofibroblastic appearance at the ultrastructural level^32^ and it was recently noted to reduce collagen fibril formation^33^. The hedgehog pathway activator GLI2 is also a suggested target of pirfenidone^34^. We observed downregulation of ERK activation by pirfenidone in TGF-β treated and non-treated mesothelioma cells. In rat proximal tubular epithelial cells pirfenidone was found to reduce ERK phosphorylation, but not p38 or Smad2 phosphorylation, which fully agrees with our current observations^35^. They are also in agreement with our previous studies indicating that mesothelioma cell migration can be reduced with MEK inhibitors^10^.

Interestingly, pirfenidone was able to significantly reduce AKT levels, likely through regulation of protein stability. In addition, pirfenidone reduced CREB phosphorylation in a time- and concentration-dependent manner. CREB activation has been linked to gremlin-1 induced proangiogenic and proinfammatory activity as well as to mesothelioma cell survival^36,37^. Although in mesothelioma cells pirfenidone was not able to reduce canonical Smad-mediated TGF-β signaling, it efficiently reduced TGF-β-mediated activation of ERK and AKT. Using inhibitors of MEK and AKT the effect of pirfenidone on mesothelioma cell proliferation was replicated. The results indicate that pirfenidone can target two major pathways, MAPK/ERK and PI3K/AKT, which are both activated in mesothelioma^7^. Our studies are in agreement with previous studies suggesting that pirfenidone can decrease EMT or myofibroblast differentiation by modulating MAPK or AKT pathways in other cell models^25,38,39^. Taken together, it is likely that multiple mechanism contribute to the effects of pirfenidone and that inhibition of non-canonical TGF-β-mediated signaling pathways plays a role in the anti-fibrotic and anti-cancer effects.

Oral administration of pirfenidone as a single agent did not reduce mesothelioma cell proliferation in a mouse xenograft model. Analyses of the effects of pirfenidone on metastasis formation was not possible in this model, since it would have taken a considerably longer follow-up time. The 200 mg/kg/day dose was selected based on previous mouse studies^40^. It is possible that a higher dose may have been effective; however, two studies using non-small cell lung cancer (NSCLC) xenograft mouse models suggested also that pirfenidone alone did not significantly reduce tumor proliferation^27,41^. In both studies combining pirfenidone with a platin chemotherapy compound resulted in a significant decrease in tumor growth. Along these lines, studies proposed that in mammary tumor models pirfenidone improves the anti-tumor efficacy of doxorubisin, likely through modulation of the tumor microenvironment e.g. by reducing collagen levels^42,43^. We hypothesize that pirfenidone in combination with chemotherapy is likely to have a similar therapeutic effect in mesothelioma. However, this remains to be demonstrated. We performed RNA sequencing of the tumors and found that pirfenidone downregulated the expression of several collagen genes as part of an extracellular matrix organization gene signature. Furthermore, the expression of *GREM1* was downregulated by pirfenidone also in *in vivo* xenograft tumors consistent with cell culture observations. These results suggest that pirfenidone can modulate the tumor microenvironment by reducing fibrotic gene expression. This may reduce the ECM barrier, which hinders drug penetration into the tumor tissue. Consistent with this, in a mammary tumor model pirfenidone decreased interstitial fluid pressure and increased blood vessel functionality leading to better drug delivery^43^.

The kinase inhibitor nintedanib is an anti-fibrotic drug also used in the treatment of IPF and was recently tested in the treatment of mesothelioma in combination with pemetrexed/cisplatin chemotherapy regiment. A phase II study suggested that addition of nintedanib improved progression free survival in malignant pleural mesothelioma patients^44^. In our study, pirfenidone was shown to reduce two major upregulated pathways in mesothelioma and to target both tumor cells and the microenvironment, which makes it a candidate for similar clinical studies. Since mesothelioma is characterized with tumor suppressor gene mutations instead of activation of “druggable” oncogenes, targeting the upregulated signaling pathways presents a therapeutic approach. In addition, chemotherapy is often associated with fibrotic alterations in the tumor tissue, which further suggests that pirfenidone may present a novel anti-fibrosis and anti-cancer adjuvant therapy for mesothelioma.

## Materials and Methods

### Reagents and antibodies

Antibodies used for Western blotting were P-Smad2 (#3108), Smad2 (#5339), P-ERK1/2 (#9101), ERK1/2 (#9107), P-AKT (#9271), P-p70S6K (#108D2) and p70S6K (#2708) from Cell Signaling, P-CREB (RLP0075) and AKT (YT0178) from Immunoway (see Supplementary Fig. 1) and tubulin loading control (ab7291) from Abcam. Antibodies used for immunohistochemistry were anti-Ki67 (Abcam, ab16667) and anti-human laminA + C nuclear envelope marker (Abcam, ab108595), which was used to identify human tumor cells in mouse tissue. Pirfenidone, cisplatin, pemetrexed, the AKT inhibitor MK-2206 and the MEK inhibitor PD98059 were from Selleckchem. Pirfenidone concentrations ranging from 100–750 μg/ml (0.54–4.04 mM) were used in experiments. The proteasome inhibitor MG132 was from Sigma-Aldrich. TGF-β1 was from R&D Systems.

### Cell culture

Human mesothelioma cell line H2052 (CRL-5915) was from American Type Culture Collection (ATCC), human mesothelioma cell line JL-1 (ACC-596) from DSMZ-Deutsche Sammlung von Mikroorganismen und Zellkulturen GmbH (Germany) and mouse mesothelioma cell line AB12 (CBA-0416) from Cell Bank Australia. JP5 mesothelioma cells were acquired from pleural effusion sample from a patient suffering from malignant mesothelioma as described^12^. Cells were cultured in RPMI-1640 medium (Sigma-Aldrich) supplemented with 10% (human cells) or 5% (mouse cells) heat-inactivated fetal bovine serum (FBS, Thermo Fisher Scientific), 1% L-glutamine, penicillin (100 U/ml), and streptomycin (100 µg/ml). Cells were incubated at 37 °C in 5% CO2.

### Proliferation assay

Cell proliferation/viability was assessed by WST-1 colorimetric assay (BioVision, Milpitas, CA). Briefly, cells were seeded into 96-well plates (5.000/well) and treated the next day with different concentrations of pirfenidone, cisplatin, pemetrexed or the inhibitors MK-2206 or PD98059 in medium containing 1% FBS. The metabolic activity of cells was measured after 48 hours according to the manufacturer’s instructions. At least three independent experiments were performed.

### Cell Migration Assays

Transwell cell migration/invasion assays were performed as described previously^11^. The inserts (8 μm pores, Corning Costar) in 12-well plates were coated on the outer surface with collagen-I (45 μg/ml, BD Bioscience) and the lower chamber was filled with serum-free medium. Cells (1–2 × 10^5^) in serum-free medium were added to the upper chamber. Pirfenidone and other agents were added to the lower and upper chambers where indicated. After an 18-hour incubation the insert filters were fixed in 4% paraformaldehyde and stained with 0.5% crystal violet (in 20% methanol). Cells on the upper side of the membrane were scraped off, and migrated cells on the lower side were photographed using AxioVert 200 microscope (Carl Zeiss) and counted. At least three independent experiments were performed.

### 3D growth and invasion assay

The 3D collagen invasion assay was performed as described^11^. Briefly, cell spheroids were formed by culturing cells in 0.5% agarose coated 96-well round bottom plate. Spheroids were then picked up, mixed with type I collagen (Sigma) and dropped on the surface of a culture plate. After collagen polymerization, normal growth medium was added. Pirfenidone or water as a control was added to the collagen matrix and growth media where indicated. Growth and sprouting of cells from spheroids was monitored and photographed using AxioVert 200 microscope (Carl Zeiss). The spheroid surface area was determined using FijiImageJ 64 bit software as described^11^. Fold change was calculated by comparing the spheroid surface area to the surface area at time point zero, which was set to one. At least three independent experiments were performed.

### TGF-β/BMP luciferase reporter assays

Cells were seeded into 96-well plates (5.000–10.000/well) prior to transfection. The cells were co-transfected with promoter constructs (CAGA)_12_-luc (Smad3 responsive) or (Bre)_2_-luc (Smad1/5 responsive), kindly provided by Dr. Peter ten Dijke (Leiden University Medical Center, the Netherlands), together with pRL-TK (Renilla luciferase control, Promega) plasmid using Fugene HD transfection reagent (Promega). After a three-day incubation, the cells were lysed and subjected to luciferase activity measurement by Dual Luciferase Reporter Assay (Promoga) and FLUOstar Omega (BMG Labtech). The luciferase activities were normalized to constitutively expressed Renilla luciferase activities.

### RNA isolation from cultured cells and quantitative RT-PCR

Total cellular RNA was isolated using RNeasy Mini kit (Qiagen) and reverse transcribed to cDNA using iScript cDNA synthesis Kit (Bio-Rad). The cDNAs were amplified using TaqMan Assays-on-Demand gene expression products (Applied Biosystems) and CFX96 Real-time PCR detection system (Bio-Rad). The relative gene expression differences were calculated with the comparative ΔΔCT method and the results are expressed as mRNA expression levels normalized to the levels of a gene with a constant expression (TBP, TATA-binding protein).

### Western blotting analysis

Western blot analyses of whole-cell protein lysates (RIPA lysis buffer supplemented with protease and phosphatase inhibitors, Thermo Fisher Scientific) were performed as described^12^. Protein concentrations were assayed using a BCA protein assay Kit (Pierce). Equal amounts of protein were separated by SDS-PAGE using 4–20% gradient Tris-glycine gels (Bio-Rad) and transferred to nitrocellulose membranes (Bio-Rad). After incubation with primary and secondary antibodies, the proteins were detected using Western Bright Quantum chemiluminescence detection system (Advansta). Scanning of gels was performed using Epson Perfection 4490 Photo Scanner, and quantification using Fiji ImageJ. In some cases, Odyssey imaging (Li-Cor Biosciences) was used and the relative band densities quantified with Image Studio Lite Ver 5.2 software (Li-Cor Biosciences).

### Human phospho-kinase array

Control and pirfenidone treated cells were lysed and alterations in phospho-kinase levels were analyzed using a Proteome profiler array (ARY003b, R&D Systems) according to manufacturer’s instructions. Quantity One version 4.6 (BioRad, Hercules, CA) was used for quantification. The kinase array was performed twice.

### ELISA assays

InstantOne ELISA kits (eBioscience) were used to detect CREB (total/phospho) or MAPK family activation (phosphorylated ERK1/2, p38 MAPK and JNK1/2) according to manufacturer’s instructions. Cells were treated with pirfenidone or other agents for 4 h in medium containing 1% FBS before lyses of cells and ELISA detection. The results are expressed relative to control. Three independent experiments were performed.

### Subcutaneous mesothelioma xenografts

All experiments involving animals were approved by the Finnish National Animal Experiment Board (ESAVI/2083/04.10.07/2015) and carried out in accordance with University of Helsinki institutional guidelines, which fulfill the requirements defined in regulations of the Finnish Act on the Protection of Animals used for Scientific or Educational Purposes (497/2013) and were performed according to the 3 R. The experiments were carried out at the University of Helsinki Laboratory Animal Center. Female 6-week-old Balb/c nu/nu mice (Scanbur) were used. The mice were randomly divided into two groups upon arrival (n = 8). JP5 cells (1 × 10^6^) expressing firefly luciferase were injected mixed with Matrigel matrix (BD Biosciences, final concentration 7 mg/ml) into the flank region of mice as previously described^11^. The procedures were performed under isoflurane anesthesia and the mice were observed until recovery. The treatment group received pirfenidone, which was started one day after cell injection (200 mg/kg in PBS, 200 μl by oral gavage) and continued daily until mice were sacrificed (60 days after cell injection). The control group received PBS only (200 μl by oral gavage). Successful injection of tumor cells as well as tumor growth was followed by bioluminescence imaging. D-Luciferin (Regis Technologies) in PBS was injected intraperitoneally and allowed to circulate for 10 min. Emitted photons were monitored with IVIS 100 whole animal imaging system (Perkin-Elmer). The weight of mice was monitored regularly throughout the experiment. Tumor tissue of sacrificed mice was collected in RNAlater solution (Life Technologies) or fixed in 4% paraformaldehyde, dehydrated and embedded in paraffin.

### Immunohistochemistry

Paraffin-embedded tissue samples were processed and stained using the Leica BOND-MAX fully automated staining system (Leica Bond Polymer Refine Detection-kit and Bond Epitope Retrieval Solution 1, 20 mins). The primary antibody incubation was 60 min. Negative control sections were incubated with rabbit isotype control (Invitrogen). The slides were scanned digitally (Pannoramic FLASH II, 3DHistech) and analyzed using CaseViewer program and HistoQuant quantification module (3DHistech).

### RNA isolation from tumor xenografts, RNA sequencing and analysis

Total cellular RNA was isolated from tumor tissue using QIAzol and RNeasy Mini kit (Qiagen). RNA integrity was analyzed using TapeStation (Agilent) at the Biomedicum Functional Genomics Unit (HiLife, Helsinki). All the RIN values of the samples were >8.0. RNA libraries were constructed using TruSeq Stranded Total RNA with Ribo-Zero H/M/R (Illumina) and RNA sequencing (75 bp, SE) was performed using NextSeq. 500 (Illumina) at DNA Sequencing and Genomics Laboratory (Institute of Biotechnology, HiLife, Helsinki). Samples with total read alignment to more than 69% human genome (ranging between 69–86%; using STAR aligner to GRCh38.90) were analyzed in Chipster v3.12 using DESeq. 2 and edgeR for differential gene expression^45,46^. Principal component analyses performed with DESeq. 2 indicated that 4/5 PFD-treated tumor samples clustered together, while 1/5 tumor sample was closer to the Ctrl-treated tumor sample cluster (n = 3). Two lists of differentially expressed coding genes (list-1: PFD-treated samples n = 4, list-2: PFD-treated samples n = 5, Supplementary Table 1) were composed of genes identified by both algorithms, showing more than −0.6 or 0.6 fold change (FC) and FDR <0.01 (edgeR). All but one gene (17/18) in list-2 were present also in list-1 (127 genes), which was used for enriched pathway analysis using Reactome^47,48^. Verification of differential gene expression of selected genes was performed using human specific TaqMan Assays-on-Demand gene expression products (Applied Biosystems).

### Statistical analyses

Comparisons were made using nonparametric tests with SPSS version 24 software (IBM). Two-group comparisons were made using Mann-Whitney U-test. P values below 0.05 (two-tailed) were considered statistically significant.

### Data availability

The RNA sequencing data have been deposited in NCBI Gene Expression Omnibus (GEO) database^49^ and are accessible through GEO Series accession number GSE113803.

## Electronic supplementary material

Supplementary figures

Dataset 1
