# Supplementary material for: Pirfenidone decreases mesothelioma cell proliferation and migration via inhibition of ERK and AKT and regulates mesothelioma tumor microenvironment in vivo
Source: Sci Rep. 2018 Jul 3;8:10070. doi: 10.1038/s41598-018-28297-x (PMC6030186; doi:10.1038/s41598-018-28297-x)

Pirfenidone decreases mesothelioma cell proliferation and migration via inhibition of ERK and AKT and regulates mesothelioma tumor microenvironment *in vivo*

Chang Li, Veronika Rezov, Emmi Joensuu, Ville Vartiainen, Mikko Rönty, Miao Yin, Marjukka Myllärniemi and Katri Koli

Supplementary Figure 1. A. P-CREB antibody recognizes the phosphorylated form of CREB as expected. Forskolin (10µM, 15 min) was used to increase CREB phosphorylation in JL-1 cells. Tubulin was used as a loading control. The molecular weight markers (kDa) are shown on the left. **B.** The AKT antibody recognizes all three AKT isoforms. CHO cells were transiently transfected with AKT1, AKT2 or AKT3 expression plasmids (addgene) and Western blotting was performed three days after transfection. Tubulin was used as a loading control. The molecular weight markers (kDa) are shown on the left.

Supplementary Figure 1.

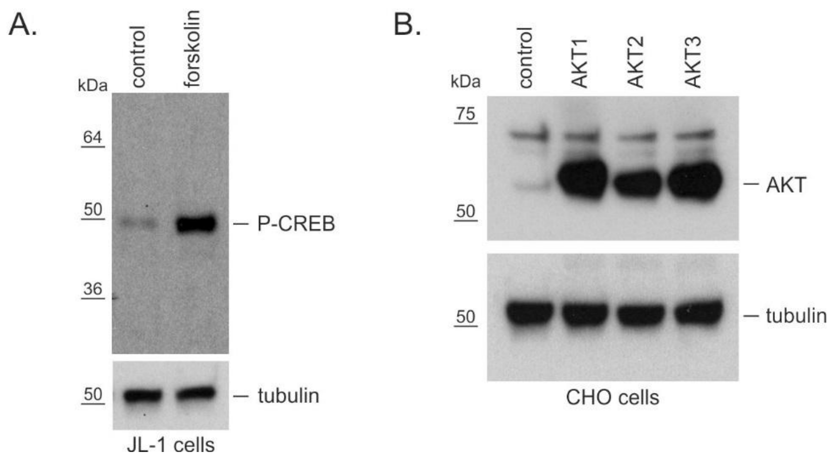

Supplementary Figure 2. Pirfenidone reduces mesothelioma cell 3D invasive growth. Invasive growth of control or pirfenidone (PFD) treated JL-1 cells was analyzed in 3D collagen 1 matrix. Representative images at time points 0h, 24h, 48h and 72h are shown.

Supplementary Figure 2.

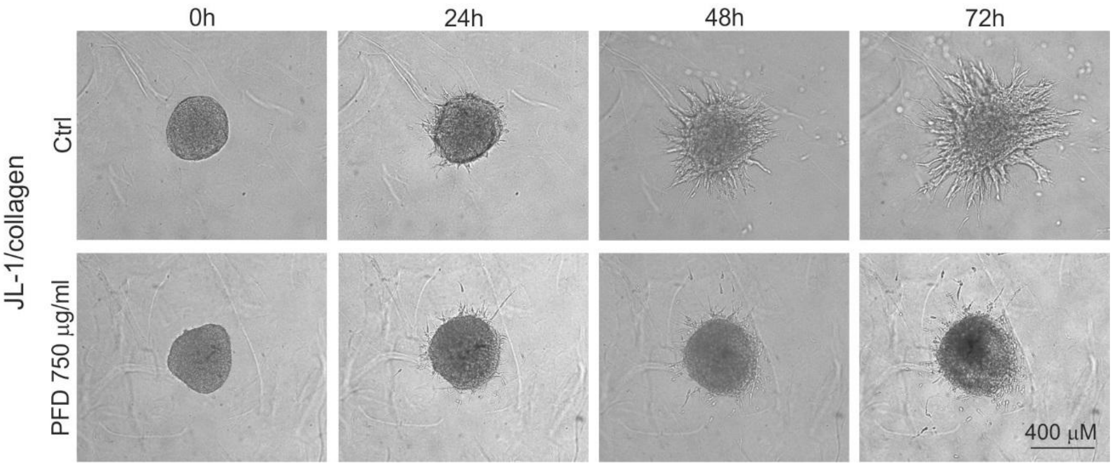

Supplementary Figure 3. P-CREB regulation by pirfenidone. Cell lysates from pirfenidone (PFD, 750 µg/ml), MG132 (10 µM) and/or TGF-β1 (0.5 ng/ml) treated (4 hours) cells were analyzed by Western blotting. Quantification of P-CREB band intensities, which were normalized using the tubulin loading control. The results are expressed relative to each control (JL-1 or H2052), which was set to 1. A representative experiment is shown.

Supplementary Figure 4. Pirfenidone induces a non-significant decrease in mouse weight. Relative weight of control and pirfenidone treated mice is shown at different time points. The error bars represent SD (n=8).

Supplementary Figure 3.

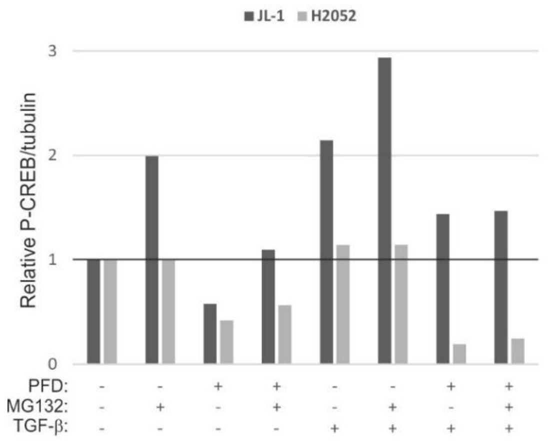

Supplementary Figure 4.

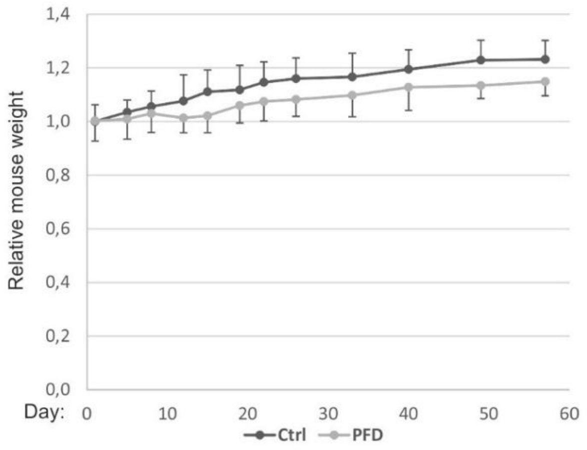

Supplementary Figure 5. Immunoblots from figure 5. A. longer exposures of P-AKT and AKT immunoblots are shown. B. Original full length Western blot images from panel D are shown. Forskolin (FSK) treated sample was used to detect P-CREB.

Supplementary Figure 5.

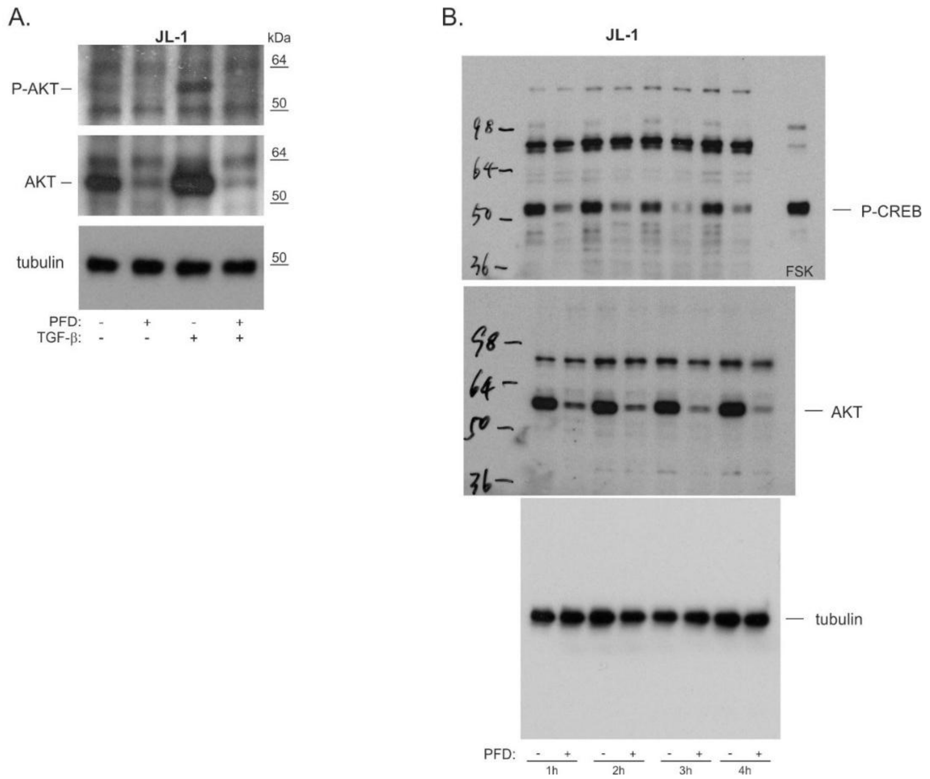

Supplement: Supplementary file 1 — Supplementary figures [file 41598_2018_28297_MOESM1_ESM.pdf]
